# RNA Pol II Promotes Transcription of Centromeric Satellite DNA in Beetles

**DOI:** 10.1371/journal.pone.0001594

**Published:** 2008-02-13

**Authors:** Željka Pezer, Đurđica Ugarković

**Affiliations:** Department of Molecular Biology, Ruđer Bošković Institute, Zagreb, Croatia; Duke University, United States of America

## Abstract

Transcripts of centromeric satellite DNAs are known to play a role in heterochromatin formation as well as in establishment of the kinetochore. However, little is known about basic mechanisms of satellite DNA expression within constitutive heterochromatin and its regulation. Here we present comprehensive analysis of transcription of abundant centromeric satellite DNA, PRAT from beetle *Palorus ratzeburgii* (Coleoptera). This satellite is characterized by preservation and extreme sequence conservation among evolutionarily distant insect species. PRAT is expressed in all three developmental stages: larvae, pupae and adults at similar level. Transcripts are abundant comprising 0.033% of total RNA and are heterogeneous in size ranging from 0.5 kb up to more than 5 kb. Transcription proceeds from both strands but with 10 fold different expression intensity and transcripts are not processed into siRNAs. Most of the transcripts (80%) are not polyadenylated and remain in the nucleus while a small portion is exported to the cytoplasm. Multiple, irregularly distributed transcription initiation sites as well as termination sites have been mapped within the PRAT sequence using primer extension and RLM-RACE. The presence of cap structure as well as poly(A) tails in a portion of the transcripts indicate RNA polymerase II–dependent transcription and a putative polymerase II promoter site overlaps the most conserved part of the PRAT sequence. The treatment of larvae with alpha-amanitin decreases the level of PRAT transcripts at concentrations that selectively inhibit pol II activity. In conclusion, stable, RNA polymerase II dependant transcripts of abundant centromeric satellite DNA, not regulated by RNAi, have been identified and characterized. This study offers a basic understanding of expression of highly abundant heterochromatic DNA which in beetle species constitutes up to 50% of the genome.

## Introduction

Satellite DNAs are tandemly repeated sequences that are in the form of long, Mb size arrays located in heterochromatin. Besides being a major constituent of heterochromatin they act as a centromere-building element in many eukaryotes [1]. Their sequence structure based often on simple repeats as well as heterochromatic localization lead to the belief that they are not transcribed [2]. However, transcripts of satellite DNAs have been reported in various species groups including vertebrates, invertebrates and plants [reviewed in 3]. In invertebrates, satellite transcripts were observed on Y chromosome loops in primary spermatocyte nuclei of *Drosophila melanogaster* [4] and *Drosophila hydei* [5]. Transcription of satellite DNAs was found often in species of Hymenoptera [6], [7]. Some satellite DNA transcripts were found exclusively in nuclei such as *D. melanogaster* and *D. hydei* Y chromosome associated transcripts [4], [5] while in most of the cases the transcripts were found as polyadenylated RNA in the cytoplasm. Transcripts exhibit high size heterogeneity and are found in some cases to be strand specific like in wasp *Diadromus pulchellus* [8], or are transcribed on both DNA strands like in ant *Aphaenogaster subterranea* [7] and wasp *Diprion pini* [6]. The existence of stage specific, differentially expressed transcripts in a number of species is consistent with their regulatory role which is however mostly not elucidated. Recently it has been shown that human satellite III transcripts, induced by stress, recruit the splicing factors to nuclear stress granules regulating in this way splicing function [9], [10].

Studies of fission yeast revealed the processing of long double strand transcripts deriving from tandemly repeated pericentromeric DNA into small 20–25 bp long RNAs involved in RNA interference (RNAi) pathway [11]. RNAi guides histone modification, in particular methylation of H3 at lysine 9 (H3K9me2) which is characteristic for heterochromatin, via an interaction between complexes containing small interfering RNAs (siRNAs) and nascent transcripts at the locus [12]. In addition to fission yeast the connection between RNAi and centromeric heterochromatin formation has been described for different systems such as plants, insects and mammals [13]. Recently it has been shown that in addition to small interfering RNAs deriving from alpha satellite DNA, long, single stranded alpha satellite DNA transcripts encompassing a few satellite monomers are functional components of the human kinetochore [14]. This alpha satellite RNA is required for the association of kinetochore proteins CENP-C1 and INCENP in the human interphase nucleolus as well as at the centromere.

Despite high evolutionary dynamics some satellite DNAs such as those belonging to the insect genus *Palorus* (Tenebrionidae, Coleoptera) are preserved and widely distributed among taxonomically distant species [15]. PRAT represents the major satellite in the species *Palorus ratzeburgii* comprising 40% of the genomic DNA and is located in regions of centromeric and pericentromeric heterochromatin on all chromosomes [16]. PRAT is distributed among all congeneric species and far beyond the level of genus in the form of low copy number satellite preserving centromeric and pericentromeric location [17]. The sequence of PRAT cloned from species that are separated by a significant evolutionary period of about 50–60 Myr showed high conservation without any fixed species specific mutation [15], [18]. It was proposed that long evolutionary preservation and sequence conservation of PRAT suggests a possible functional significance.

Most of current research is focused on the explanation of role of satellite DNA transcripts but little is known about the transcription itself although it is likely to be the key regulatory step in biogenesis of functional transcripts. In the case of fission yeast *Schizosaccharomyces pombe* it is shown that RNA pol II promotes transcription of centromeric DNA [19]. Transcription of γ satellite DNA which is a major constituent of mouse pericentromeric heterochromatin is also mediated by RNA pol II [20]. The aim of this study is to characterize transcripts of abundant centromeric satellite DNA of beetles and to identify the basic mechanism of satellite DNA transcription. This can help to understand the mechanisms of regulation of expression of highly abundant satellite DNA sequences which in beetle species constitute up to 50% of the genome.

## Materials and Methods

### RNA isolation

Total, cytoplasmic and nuclear RNAs for the purpose of RT-PCR, real-time PCR, Northern and RLM-RACE analysis were isolated using the Rneasy Mini Kit (Qiagen), whereas polyadenylated RNA was recovered on Oligotex columns (Qiagen). RNA for primer extension reaction as well as RNA to be used for detection of siRNAs on Northern blots were extracted by phenol-chloroform [21]. RNA preparations were enriched for small-sized RNA by removing high molecular weight molecules by precipitation with 5% polyethylene glycol and 0.5 M NaCl [22]. To eliminate traces of co-extracted DNA all preparations were treated with up to 0.5 units of DNase I per microgram of RNA prior to further reactions.

### RT-PCR

20 ng of RNA was transcribed into DNA and amplified using the OneStep RT-PCR Kit (Qiagen) and primers RATZEB1 (5′-GAATCCGAAACAAAATCGTGCC-3′) and RATZEB2 (5′-GCTGAAATCTTGCAAACTTTAC-3′) specific to the PRAT. To investigate strand origin of transcripts, reverse transcription was performed at 50°C for 30 min with one primer only, followed by heat inactivation of reverse-transcriptase and activation of HotStarTaq DNA Polymerase for 15 min at 95°C. At this step the second primer was added to start 33 cycles of PCR under the following conditions: 1 min at 94°C, 30 s at 58°C and 1 min at 72°C. Final extension was done for 10 min at 72°C. Reactions in which reverse transcription was omitted as well as reactions with no added template were performed as controls for DNA contamination and cross-contamination between samples.

### Northern and dot blot analysis

RNA was separated on 1.2% agarose-formaldehyde gels and blotted onto positively charged nylon membranes. For small RNA analysis, 30 µg of RNA enriched for low molecular weight molecules was resolved on 12% polyacrylamide gels containing 8 M urea and transferred to nylon membranes in 0.5× TBE for 45 min at 100 V using wet electroblotter (BioRad). In dot blot analysis, samples of RNA were applied to membrane together with the equal amounts of yeast RNA which served as a control for hybridization specificity. Unlabelled “run off” transcripts produced in vitro from vector containing PRAT dimer sequence were used as positive controls and for calibration after hybridization. Hybridization was performed under high stringency conditions at 50°C and 60°C on dot blots and high molecular weight RNAs, and at 22°C and 40°C on blots with small RNAs, in 10 ml solution containing 50% formamide, 5× Denhardt solution, 6× SSPE, 0.1% SDS, 100µg/ml herring sperm DNA, and 1-2×10^7^ cpm of DNA probe labelled by random priming or in vitro transcribed RNA probe. To produce strand specific RNA probes, pGEM-T vector (Promega), with previously cloned PRAT dimer, was digested with *Nco*I or *Sal*I and in vitro transcribed by T7 or SP6 RNA polymerase, respectively, in a reaction containing [α-^32^P]UTP. After hybridization, membranes were washed twice in 2× SSC, 0.1% SDS at RT, once in 0.1× SSC, 0.1% SDS at 37°C, and exposed overnight on a phosphor-imager or up to 7 days on autoradiography films. Relative amounts of PRAT transcripts were determined by densitometry, subtracted for the signal corresponding to yeast RNA.

### Primer extension

Four oligonucleotides, RATZEB1, RATZEB2, RATZEB3 (5′-GCTGTATTTAAG AGAATCCG-3′) and RATZEB4 (5′-ATACAGCTGAAACCATGC-3′) corresponding to PRAT sequence were end-labelled with [γ-^32^P]dATP in phosphorylation reaction with T4 polynucleotide kinase (Fermentas). Each of the labelled primers was incubated with total RNA (10–30 µg) at 65°C for 90 min and slowly cooled down to RT to allow hybridization of primer to RNA. 25 U of AMV reverse transcriptase (Roche) was added to 40 µl reaction and incubated at 42°C for 1 h. The reaction was terminated by incubation with RNase A at 37°C for 30 min, and reverse transcripts were recovered by phenol-chloroform extraction and ethanol precipitation. Primer extension products were electrophoresed through a 6% denaturing polyacrylamide gel along with the sequencing products of PRAT dimer clone obtained with the same labelled oligonucleotide used for primer extension.

### RNA Ligase Mediated Rapid Amplification of cDNA Ends (RLM-RACE)

In order to identify specific 5′ capped transcripts, potentially transcribed by RNA polymerase II, a 5′ RACE reaction was conducted on total RNA from larvae according to manufacturer's instructions (Ambion). After phosphatase and pyrophosphatase treatment, the RNA adapter was ligated to the 5′ end of decapped target molecules. RT-PCR was performed using the OneStep RT-PCR Kit as described above, with an outer adapter-specific primer and RATZEB1 or RATZEB2 primer. Additional PCR on obtained products was conducted with either RATZEB1 or RATZEB2 satellite specific primer, and inner adapter-specific primer to increase specificity of amplification. Simultaneous experiment without pyrophosphatase treatment (-TAP) served as a negative control. The same kit was used for 3′ RACE reactions to detect transcription termination sites. Total RNA from larvae was reverse transcribed using the supplied 3′ RNA adapter which has an oligo T stretch, and subsequently amplified using adapter specific outer primer and RATZEB1 or RATZEB2 primer.

All PCR products from both 5′ and 3′ RACE were cloned in the pGEM-T Easy vector (Promega), positive clones were screened by colony lift and hybridization with PRAT probe, followed by sequencing.

### Cloning and sequence analysis

A primer extension product of 122 nt size, obtained in reactions with RATZEB2, was amplified by using RATZEB1 and RATZEB2 primers. Amplified PCR product of 131 bp was directly sequenced using RATZEB2 as a sequencing primer. The same amplification product was ligated into the pGEM-T vector and transformed into *E. coli*. Eleven clones were randomly chosen and sequenced in both directions using vector-specific primers. Accession numbers of PRAT RNA monomers obtained by direct sequencing and cloning are EF209021-EF209033. Nucleotide sequences were aligned and compared using ClustalW. Putative RNA Polymerase II promoter site was annotated using Neural Network Promoter Prediction Tool (http://www.fruitfly.org/seq_tools/promoter.html; 23).

### Injection of larvae

Late instar larvae of *P.ratzeburgii* were cooled down on ice and fixed onto a double-stick tape placed over a micro slide. Larvae were injected dorsally into the hemolymph using a simple injection apparatus [24]. 23–26 larvae were injected with 0,1–0,15 µl per larvae of injection buffer (1,4 mM NaCl; 0,07 mM Na_2_HPO_4_; 0,03 mM KH_2_PO_4_; 4 mM KCl; 5% green food dye) or alpha-amanitin, 1 µg/ml and 10 µg/ml, diluted in injection buffer. After injection larvae were removed from the tape and grown on whole wheat flour at 30°C for five days. No mortality or toxic effects were noted for larvae treated for five days with such concentrations of alpha-amanitin. The low mortality was observed for larvae injected with 100 µg/ml of alpha-amanitin.

### Real-time PCR

0.5 µg of total RNA isolated from injected larvae was reverse transcribed by 20 U of M-MuLV Reverse Transcriptase (Fermentas) using random primers in a final volume of 20 µl. Resulting cDNA (20–40 ng per reaction) was subjected to real-time quantitative PCR analysis. Primers and TaqMan probes for PRAT as well as for 18S rRNA (endogenous control) were obtained from Applied Biosystems as TaqMan Gene Expression Assays. All reactions were performed on ABI 7300 Real-Time PCR System in four replicates in a final volume of 50 µl, under following reaction conditions: pre-incubation at 50°C for 2 min, 95°C for 10 min and 40 cycles of 95°C for 15 sec and 60°C for 1 min. Non-template controls were included. The data were analyzed using the comparative C_T_ method (ΔΔ C_T_). cDNA from larvae injected with buffer served as calibrator and 18S rRNA gene was used to normalize PRAT quantities.

## Results

### PRAT transcripts are heterogeneous in size and differentially expressed from both strands

**Figure 1:**
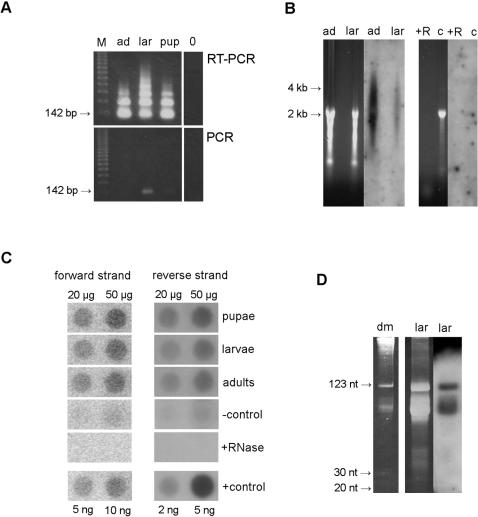
Detection of PRAT satellite transcripts. *(A)* RT-PCR with primers RATZEB1 and RATZEB2 on total RNA from adults (ad), larvae (lar) and pupae (pup). For each reaction PCR was conducted in parallel without prior reverse transcription as a control for genomic DNA contamination, while 0 is control with no template. M is size standard. *(B)* Total RNA separated on 1.2% denaturing agarose gel (18 µg and 13 µg from adults and larvae, respectively) and corresponding Northern blot after hybridization with ^32^P labelled PRAT DNA probe. 18 µg of adult RNA digested with RNase A represents control for residual genomic DNA (+R) while 18 µg of *D. melanogaster* RNA (c) is used as a negative control. *(C)* Dot blot analysis of total RNA, using ^32^P labelled RNA probes for forward and reverse strand, respectively. 20 µg and 50 µg of larval RNA digested with RNase A represent control for genomic DNA contamination (+RNase) while the same amounts of *S. cerevisiae* RNA are used as a negative control (−control). Complementary transcripts, in amounts indicated, are used as a positive control (+control) and for calibration. *(D)* 30 µg of larval low molecular weight RNA (lar) separated on 12% polyacrylamide gel and Northern blot analysis with RNA probe for reverse strand, labelled with ^32^P. Hybridization was performed at 22°C and blot was exposed for one day on autoradiography film. Longer exposition as well as rehybridization with RNA probe for forward strand did not reveal signal in the siRNA region. *D. melanogaster* RNA (dm) serves as a size marker.

RT-PCR with primers specific for PRAT satellite DNA revealed the presence of transcripts in total RNA isolated from *P. ratzeburgii* adults, larvae and pupae (Fig. 1a). The amplified fragments are of expected size corresponding to 142 bp PRAT satellite monomer and its multimers, indicating that transcripts do not terminate within each monomer but are composed of regularly arranged tandem repeats characteristic for PRAT satellite DNA. To ensure that the amplification results are from reverse transcribed RNA and not from residual genomic DNA, each reaction was conducted simultaneously without prior reverse transcription. Similar amplification profiles were obtained using primers complementary to either forward or reverse strand in reverse transcriptase reaction indicating that transcription of PRAT proceeds from both DNA strands.

Transcripts were further characterized by Northern hybridization using DNA probe labelled with ^32^P (Fig. 1b). Total RNA was isolated from three developmental stages, pretreated with DNase I, and hybridization was performed under high stringency conditions. The hybridization signal appears as a smear ranging from approx. 0.5 kb up to 5 kb without any distinct bands. The heterogeneous size profile of transcripts might result from their rapid processing or could be due to multiple initiation and termination sites. The hybridization profiles were very similar in all three developmental stages (hybridization for pupae not shown). To eliminate the possibility that the signal originates from highly abundant satellite DNA instead of RNA, the RNA samples were digested with RNase A and run on the gel as a control. No signal was detected on RNase A treated samples (Fig. 1b).

Dot blot analysis of *P. ratzeburgii* RNA using strand specific RNA probes labelled in vitro by ^32^P, revealed different quantities of transcripts from opposite DNA strands: satellite RNA corresponding to forward strand accounts for 0.03% of total RNA, while transcripts corresponding to the reverse strand constitute 0.003% of total RNA (Fig. 1c). Comparison of signals among different developmental stages revealed a similar level of PRAT expression in larvae and adults while the amount of PRAT transcripts from both strands was approx. 20% higher in pupae, indicating slight developmental regulation.

The simultaneous presence of forward and reverse transcripts may activate the RNA interference (RNAi) pathway and produce small interfering RNAs (siRNA). To verify whether the PRAT transcripts were processed into siRNA, Northern blot hybridization using RNA preparations enriched for small RNA was performed (Fig. 1d). We were unable to detect PRAT satellite DNA-specific siRNA (21–25 nucleotides) deriving either from forward or reverse strand, despite using large quantities of enriched small RNA and employing sensitive hybridization conditions at 40°C. Lowering of stringency conditions for hybridization by reducing the hybridization temperature to 22°C also did not reveal the presence of siRNAs. These results indicate that PRAT is not processed using the RNAi mechanism or that siRNA are very rare, below the level of detection.

### PRAT transcripts are located both in the nucleus and in the cytoplasm and are partially polyadenylated

**Figure 2:**
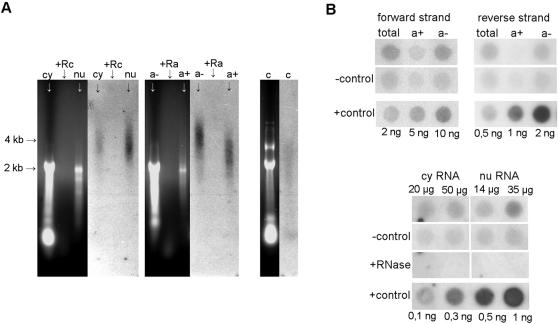
Subcellular localization and polyadenylation of transcripts. *(A)* Agarose gel electrophoresis of cytoplasmic (cy), nuclear (nu), polyadenylated (a+) and non-polyadenylated (a-) RNA and hybridization profile obtained with RNA probe for forward strand. 13 µg of cy and 1.3 µg of nu RNA extracted in parallel from the same sample of adults are loaded as well as 1 µg of a+ and 17 µg of a-. Cytoplasmic (13 µg) and non-polyadenylated RNAs (17 µg) treated with RNase A (+Rc and +Ra, respectively) serve as controls for genomic DNA contamination. *S. cerevisiae* RNA (c) represents negative hybridization control. *(B)* Dot blot analysis of total RNA (45 µg), polyadenylated RNA (a+, 6 µg), non-polyadenylated RNA (a-, 40 µg) using PRAT RNA probes for forward and reverse strand, as well as of cytoplasmic (cy) and nuclear (nu) RNA, in amounts indicated, using RNA probe for reverse strand. *S. cerevisiae* RNA is used as a negative control in amounts exactly matching those of above mentioned samples (−control). For the purpose of PRAT transcripts quantification in different RNA fractions, signal intensity was reduced for the signal detected in the corresponding negative control. Due to the low signal intensity in the a+ fraction of reverse strand precise quantification was not possible. Complementary PRAT transcripts, in amounts indicated, are used as a positive control (+control) and for calibration. Cytoplasmic and nuclear RNA digested with RNase A represent control for genomic DNA contamination (+RNase).

Subcellular localization may provide clues to the function of satellite DNA transcripts. Therefore, nuclei were separated from cytoplasm of *P. ratzeburgii* larvae, and RNA was extracted from each fraction. In addition, polyadenylated RNA was separated from total RNA on Qiagen Oligotex columns and the remaining non-polyadenylated fraction was recovered. All RNA isolations were treated with DNase I to eliminate traces of genomic DNA and subjected to Northern blot hybridization using ^32^P strand specific RNA probes (Fig. 2a). Hybridization size profiles obtained by both RNA probes were highly similar. Hybridization signals in the form of smears in the nuclear and cytoplasmic fraction are predominantly localized between 2 and 5 kb. In the non-polyadenylated fraction the majority of the signal is around 5 kb, while the polyadenylated RNA fraction is characterized with a hybridization smear of lower size, with maximal intensity around 3 kb.

In order to determine relative distribution of PRAT transcripts among different RNA fractions dot-blot hybridization was performed (Fig. 2b). Nuclear and cytoplasmic RNA fractions were isolated in parallel from the same sample of adult insects as well as polyadenylated and non-polyadenylated RNA. Comparison between hybridization signals revealed that the majority of PRAT transcripts, representing approximately 80% of the forward strand, are not polyadenylated, while the remaining 20% have poly(A) tails. Since expression of the reverse strand is 10 times lower relative to the forward one, the signal intensity was very low and not suitable for precise quantification. The comparison of hybridization signals between cytoplasmic and nuclear fractions reveal preferential localization of PRAT transcripts in the nucleus where approx. 80% of them are detected relative to 20% found in cytoplasm (Fig. 2b).

### Multiple transcription initiation are mapped within the PRAT monomer

**Figure 3:**
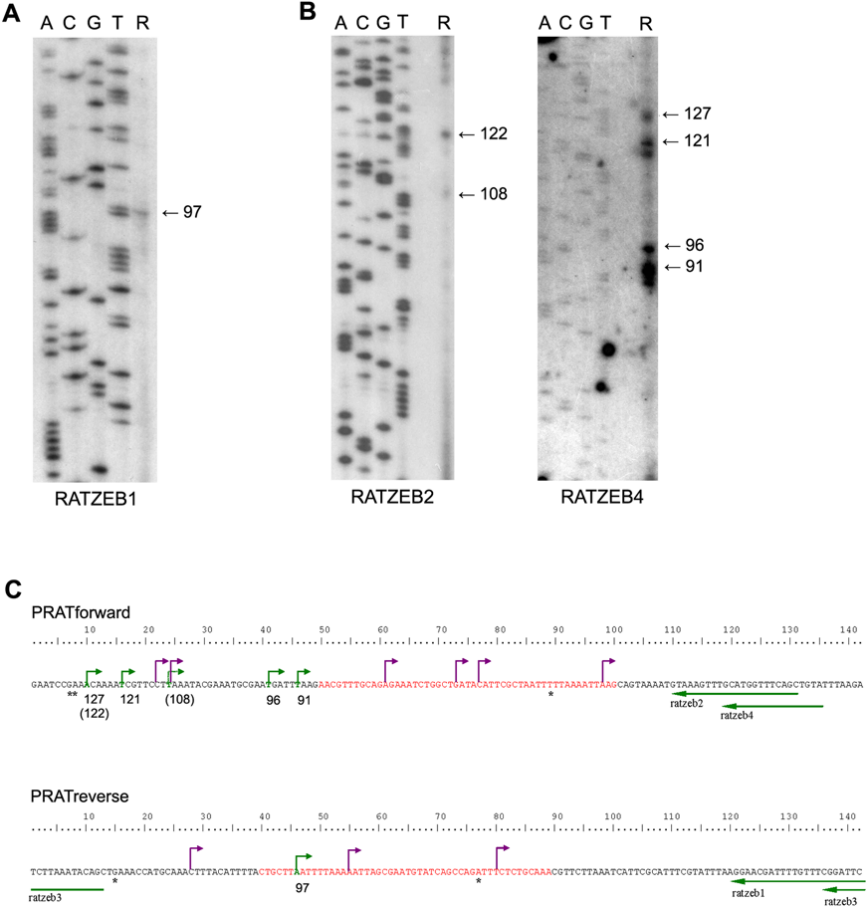
Primer extension analysis of PRAT satellite transcripts. *(A)* Single extension product is detected on reverse PRAT strand using RATZEB1 primer (line R). The size of the product of 97 nt is determined by comparison with the sequencing reaction of PRAT dimer obtained by the same RATZEB1 primer. *(B)* Several extension products are detected on the forward strand using primers RATZEB2 and RATZEB4, and their size is indicated (lines R). Products of 122 nt and 127 nt, obtained in a reactions with RATZEB2 and RATZEB4, respectively, correspond to the same start site. *(C)* Positioning of transcription start sites and polyadenylation sites on PRAT forward and reverse strand. Transcription start sites determined by primer extension are indicated by green bent arrows while those detected by 5′ RLM-RACE are shown by violet bent arrows. Dashed green arrow represents weaker start site corresponding to 108 nt band obtained in reaction with RATZEB2. The sizes of the products obtained in extension assays are indicated under the corresponding nucleotide positions. The lengths of the products from reactions with RATZEB2 are in brackets. Polyadenylation sites are indicated with asterisks. Straight arrows represent primers and are positioned under the sequence they anneal to. Position of putative polymerase II promoter predicted by a neural network method (Reese 2001) is marked in red.

A primer extension analysis was used for mapping the transcription initiation sites on PRAT satellite repeats. Oligonucleotides 5′-labelled with 32P were hybridized individually to total *P. ratzeburgii* RNA to initiate reverse transcription either from forward or reverse strand. Extended products were visualized on polyacrylamide gel and compared with satellite DNA sequence ladder obtained by the same primer on cloned satellite dimer. Primer extension using primer RATZEB1 revealed a single transcription start site on PRAT reverse strand (Fig. 3a) corresponding to position 46 on PRAT consensus sequence (Fig. 3c). This initiation site was confirmed by another primer RATZEB3 which hybridizes to PRAT upstream from RATZEB1 (not shown, Fig. 3c). Extensions on forward strand using primer RATZEB2 gave as a product strong band of 122 nt and a 108 nt band of low intensity (Fig. 3b), which correspond to positions 10 and 24, respectively, in the PRAT sequence (Fig. 3c). The same, strong initiation site at position 10 was also confirmed by primer RATZEB4 which is positioned upstream of the RATZEB2 (band 127, Figs.3b, 3c). However, primer RATZEB4 revealed three additional strong bands (Fig. 3b) which are also indicated as potential transcription initiation sites on forward strand at positions 16, 41 and 46 (Fig.3c).

In order to confirm that distinct bands obtained by RT extension derive specifically from PRAT transcript we have performed PCR amplification of 122 nt band obtained by extension with RATZEB2 primer, using standard PRAT specific internal primers RATZEB1 and RATZEB2 for PCR reaction. A 131 nt long amplified product was directly sequenced using RATZEB2 as a sequencing primer. The same PCR product was cloned into an AT vector and 11 clones were subsequently sequenced. Sequence analysis revealed high homology and correspondence to randomly cloned monomers of PRAT satellite DNA, previously determined (not shown).

**Figure 4:**
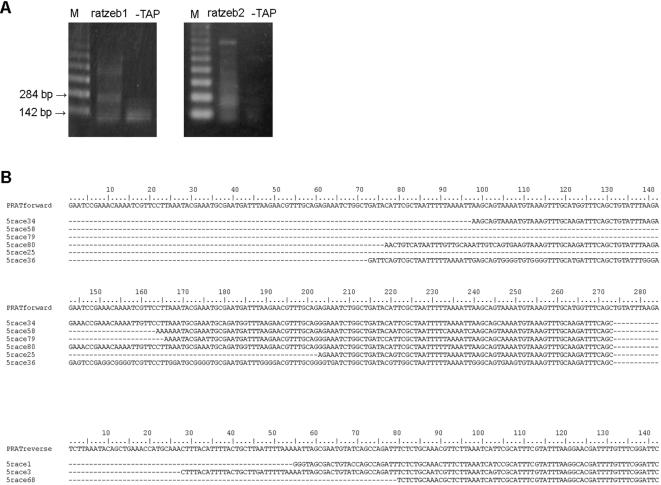
Mapping of transcription start sites by 5′ RLM-RACE. *(A)* RT-PCR products of reactions with either RATZEB1 (left) or RATZEB2 (right) primer, resolved on agarose gel. –TAP lanes represent corresponding negative controls obtained without pyrophosphatase treatment. M is size standard. *(B)* Sequences of cloned 5′ RACE products obtained with RATZEB2 are aligned with forward strand of consensus PRAT dimer (above). Sequences obtained with RATZEB1 primer are compared to reverse strand of consensus PRAT monomer (below). Missing parts of sequences are marked by a dashed line. Accession numbers of sequences obtained by 5′RLM-RACE are EF209039-EF209047.

Mapping of the transcription start sites was also performed using RLM-RACE which involves ligation of a defined RNA adapter to the 5′ ends of full length mRNA followed by RT-PCR using primers RATZEB1 and RATZEB2 for mapping the initiation sites on PRAT reverse and forward strand respectively. Products of amplification (Fig. 4a) were cloned into an AT vector, positive clones detected by hybridization and sequenced. All six positive clones obtained with the RATZEB2 primer have adapter ligated directly on the forward strand of PRAT satellite (Fig. 4b) but at different positions: 22, 24, 61, 73, 77 and 98 (Fig 4b, Fig. 3c). Among them only position 24 overlaps with the initiation site mapped by primer extension (108 nt band in Fig. 3b). The lengths of clones range from 71 nt to 201 nt, corresponding to amplification of satellite monomer and dimer (Fig. 4b). Although longer, multimeric stretches of tandem repeats were amplified (Fig. 4a) and subjected to ligation, they were not detected as positive clones, probably due to inefficient cloning and/or instability of clones. Using the RATZEB1 primer for mapping the initiation sites on the reverse strand, three clones were identified (Fig. 4b). Each clone corresponds to a different initiation site located at positions 28, 55 and 80 (Fig 4b, Fig. 3c). Neither of these sites overlaps with the single initiation site on the reverse strand as determined by primer extension.

### Multiple transcription termination sites exist within PRAT

**Figure 5:**
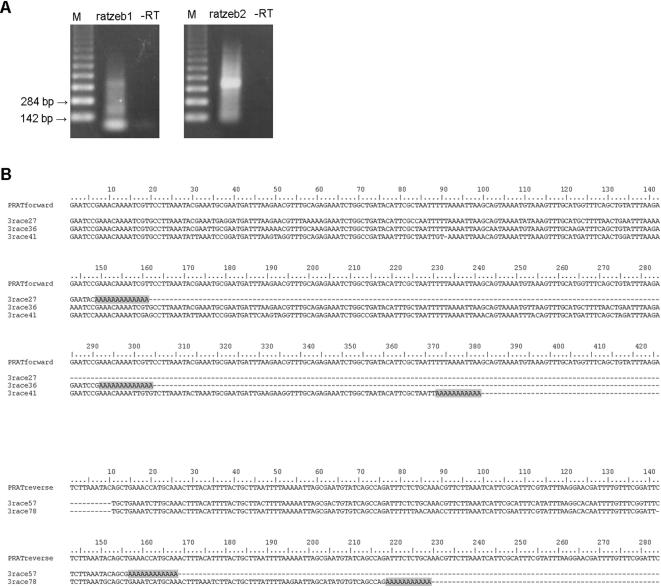
Mapping of polyadenylation sites by 3′ RLM-RACE. *(A)* PCR products of reaction with RATZEB1 (left) and RATZEB2 (right) resolved on agarose gel. –RT lanes represent corresponding negative control reactions in which reverse transcription was omitted. *(B)* Sequences of cloned 3′ RACE products obtained with RATZEB1 are shown above, aligned with forward strand of consensus PRAT trimer. Products of reaction with RATZEB2, depicted below, are compared to reverse strand of consensus PRAT monomer. Poly(A) tails are marked with grey shading. Missing parts of sequences are marked by a dashed line. Accession numbers of sequences obtained by 3′RLM-RACE are EF209034-EF209038.

Transcription termination sites were mapped using 3′ RACE. Reverse transcription was performed using the 3′ RACE adapter which has an oligo T stretch, followed by PCR amplification with satellite specific primer RATZEB1 or RATZEB2, and primer homologous to the adapter. Amplification reactions of forward and reverse strand (Fig. 5a) were randomly cloned into the AT vector and positive clones detected by hybridization. Sequences of three clones corresponding to the forward strand encompass satellite monomer, dimer and trimer, respectively (Fig. 5b). All three have termination sites within the satellite, two polyadenylation sites are mapped at positions 6 and 7 while the third one is located at position 88 of the forward strand (Fig. 5b, Fig. 3c). Since position 7 in the PRAT sequence is immediately followed by a stretch of A nucleotides it is possible that it acts as a false poly(A) tail during reverse transcription. Two clones corresponding to the reverse strand (Fig. 5b) also end within satellite DNA, having poly(A) tails at positions 15 and 76 respectively (Fig. 5b, Fig. 3c).

As in the case of 5′ RACE assay, all positive clones detected in 3′ RACE analysis correspond to shorter fragments, up to trimer, although longer multimers have been subjected to ligation (Fig. 5a). This again suggests low efficiency of cloning of longer multimers and/or instability of clones.

### Transcription of PRAT satellite is sensitive to alpha-amanitin

**Figure 6:**
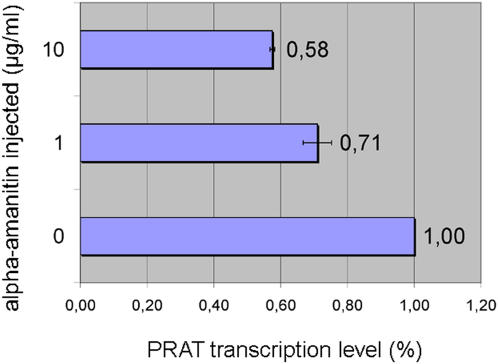
Sensitivity of PRAT transcription to alpha-amanitin. Results of quantitative real-time PCR analysis are shown. Transcription level of PRAT after injection with two concentrations of inhibitor (1 µg/ml and 10 µg/ml) is compared to the transcription level of PRAT from larvae injected with buffer. 18S rRNA gene was used as endogenous control to normalize PRAT quantities. Error bars represent standard deviations from two different real-time PCR experiments.

The presence of a cap structure as well as poly(A) tails in at least a portion of the transcripts indicates that RNA polymerase II could be responsible for PRAT satellite DNA transcription. In order to test this assumption we injected beetle larvae with alpha-amanitin, which is known to inhibit RNA polymerase II completely at low concentration, while RNA polymerase I activity remains unaffected. Larvae were injected with approx. 0.1 ng and 1 ng of alpha-amanitin respectively, and these concentrations of alpha-amanitin caused no mortality or toxic effects. Five days after injection total RNA was isolated and treated with DNase I to remove traces of genomic DNA. RNA quality was checked by electrophoresis and cDNA library was generated. Real-time PCR analysis evidenced approximately 29% and 42% reduction in PRAT transcript quantity in larvae injected with 0.1 ng and 1 ng of alpha-amanitin, respectively (Fig. 6). 18S rRNA is known to be transcribed by pol I and its transcription was unaffected by inhibitor concentrations applied here, thus it was considered convenient for endogenous control in real-time PCR.

## Discussion

The results presented in this paper reveal that evolutionarily conserved PRAT satellite DNA is continuously expressed during all three developmental stages in the beetle *P. ratzeburgii*: larvae, pupae and adults, at a similar level. PRAT is predominantly transcribed from one strand and the transcripts are relatively abundant comprising 0.03% of total cellular RNA while expression of the opposite strand is 10 times lower. The transcripts are heterogeneous with the size profile ranging from approx. 0.5 kb to more than 5 kb, matching those characteristic for the centromeric, 180 bp satellite of *Arabidopsis thaliana* [25]. However, unlike *Arabidopsis* or rice centromeric satellites whose primary transcripts are regulated by RNAi and corresponding siRNAs are readily proved by Northern hybridization [25], [26], we were unable to detect PRAT satellite-specific siRNA despite using large quantities of enriched small RNA and employing sensitive hybridization conditions. Lowering of stringency conditions for hybridization also did not reveal any presence of siRNAs. The siRNA may be extremely rare or produced in specific tissues and therefore undetectable in RNA isolated from the whole specimen. Alternatively, a distinct mechanism of heterochromatin formation which does not rely on siRNA might be operating in beetles.

Although the RNAi pathway plays a role in heterochromatin establishment in various organisms, it does not seem to be indispensable for assembly and maintenance of heterochromatin, as shown for heterochromatin initiated in mammalian cells by transgene tandem repeats [27]. Transcription of major (γ) mouse satellite DNA located within pericentromeric heterochromatin is cell cycle-regulated and proceeds in the form of small, approximately 200 nt long RNA during mitosis while abundant, long, heterogenous transcripts are induced in G1 phase [20]. However, no evidence for siRNA-sized molecules at any time during the cell cycle exists, pointing to the difference in heterochromatin expression and establishment between mammals and fission yeast. In addition, numerous examples illustrate the involvement and importance of longer RNAs for kinetochore formation. RNA encoded by centromeric satellite DNA and retrotransposons, ranging in size between 40 and 200 nt, has been shown to be an integral component of the kinetochore in maize, tightly bound to centromeric histone H3 [28]. Murine minor satellite DNA associated with the centromeric region is transcribed from both strands and transcripts are processed into 120 nt RNA which localizes to centromeres [29]. The accumulation of satellite transcripts is impaired by mislocalization of centromere-associated proteins essential for the formation of centromeric heterochromatin. In addition, long transcripts of alpha satellite DNAs have been shown to be functional component of kinetochore participating in recruitment of kinetochore proteins [14]. These results indicate that in addition to RNAi moderating heterochromatin establishment in different systems an alternative mechanism involving longer RNA operates in kinetochore establishment.

Transcription of satellite DNA might be initiated from upstream promoters provided by mobile elements inserted near satellite DNA clusters [25], [28]. However, there are cases when transcription of satellite DNA proceeds from internal promoters such as schistosome satellite having a functional RNA pol III promoter [30], or newt satellite II with small nuclear RNA (snRNA) promoter that is responsible for RNA pol II transcription [31]. Primer extension (PE) and RLM-RACE were used for mapping transcription initiation sites of PRAT transcripts. The results revealed the presence of multiple initiation sites on both strands, all of them located within the satellite monomer. On the forward strand four major and one minor initiation sites were mapped by PE, while RLM-RACE disclosed the presence of five additional start sites. On the reverse strand a single initiation site identified by PE and three additional sites detected by RLM-RACE were mapped. All transcription start sites are probably not used with the same frequency and it is reasonable to propose that those detected by the not very sensitive method of primer extension are used most often. Multiple transcription initiation sites are characteristic for «slippery promoters», a common promoter type found in TATA-box-containing and TATA-less genes of *Drosophila*, located either in heterochromatin or euchromatin [32].

The presence of a cap structure as well as poly(A) tails in a portion of the transcripts indicate that pol II could be responsible for PRAT satellite DNA transcription. The sensitivity of PRAT transcription to alpha-amanitin at concentrations that selectively inhibit pol II confirms this assumption. However, since a considerable amount of transcripts does not contain poly(A) tail, the possibility exists that RNA polymerase(s) other than pol II might also be involved in transcription. In the yeast *Schizosaccharomyces pombe* RNA polymerase II is responsible for transcription of pericentromeric regions which is coupled with RNAi-dependent heterochromatin assembly [19]. In addition, at the centromere of *S. pombe* association of RNA Pol III as well as Pol III transcripts of tRNA genes were identified [33], [34]. The examination of PRAT sequence for the presence of A and B box consensus sites [35] which are associated with RNA Pol III transcription was performed. Although no motifs of complete homology to either A or B box were found in PRAT consensus sequence, such motifs can be created by either a single point mutation (A box at position 122 of forward strand) or by two point mutations (B box at position 3 of forward strand). So, it can be proposed that some PRAT satellite monomers could have consensus sites for A and B boxes and are potentially transcribed by RNA Pol III.

We did not experimentally determine the position of promoter elements but a putative RNA polymerase II promoter site was annotated by a computational approach, using a time-delay neural network method [23]. The putative promoter element is detected with a high score of 0.95 in 52% of PRAT DNA monomers as well as in 80% of PRAT RNA monomers, between positions 40–89 nt of reverse strand (Fig. 3c), overlapping exactly with the most conserved part of the PRAT sequence, as previously revealed by sequence comparison [18]. In a few monomers a putative promoter element is annotated with score of 0.65 between positions 50 and 100 nt in the forward strand (Fig. 3c), overlapping with the conserved part of PRAT sequence. Localization of a putative promoter within the conserved part of the PRAT satellite monomer sequence might indicate possible influence of selection pressure to preserve transcriptional activity of satellite DNA. However, the possibility that transcription proceeds from a promoter located upstream of satellite clusters can not be completely excluded.

A motif exhibiting perfect homology to the TATA-box was not detected in the PRAT consensus sequence, but can be created by a single point mutation of a motif (TATTTAA) located at position 134 on the forward strand. Due to satellite sequence variability it is probably present in many PRAT monomers. Complementary sequence on opposite DNA strand (TTAAATA) also resembles TATA-box. These TATA-box like motifs are positioned 11 and 18 nt upstream of the most proximate transcription start site detected on forward and reverse strand, respectively. Overlapping of TATA-box like sequences on both DNA strands and close position of transcription initiation sites indicates bidirectional transcription from the same, central promoter.

RACE analysis revealed the presence of multiple termination sites located within the PRAT sequence. The method specifically picks transcripts ending with poly(A) tails. Some of them could result from polyadenylation, while due to the presence of long A stretches in PRAT sequence it is possible that some of the analyzed transcripts have a false poly(A) tail. In any case, the length of poly(A) tail is relatively short, below 13 nt, similar to those found in rice satellite DNA transcripts [26]. A polyadenylation signal (AATAAA) can be created at position 94 of forward strand of PRAT consensus sequence by single point mutation, as well as at position 50 of the reverse strand by two mutations. Based on multiple, irregularly distributed transcription start and stop sites detected within PRAT sequence it can be concluded that they are the major cause of the observed size heterogeneity of transcripts. Although PRAT transcripts are large in size, up to 5 kb, RACE assay did not produce stretches of tandem repeats longer than satellite trimer what can be explained by inefficient cloning of long multimers as well as by their instability in plasmid. The rearrangement and instability of long satellite multimers cloned into plasmid vectors has been previously observed for PRAT satellite DNA and is probably characteristic of many other satellite DNAs. General lack of satellite DNA transcripts in cDNA libraries of different species is most probably related to their tandem repeat substructure which is not stable in most plasmid vectors.

The function of PRAT transcripts remains highly speculative. Their preferential location in the nucleus might indicate close association with chromosomes and possible influence on chromatin modulation and centromere function. On the other hand, a small portion of PRAT transcripts which are located in the cytoplasm could perform an additional, yet unknown role. The presence of transcription initiation sites within the PRAT sequence as well as a putative promoter for RNA polymerase II point strongly to the functional significance of this evolutionary extremely conserved sequence and transcripts deriving from it. Identification of proteins, either nuclear or cytoplasmic, which interact with long PRAT satellite transcripts, could shed more light on the role of this abundant non-coding RNA and explain its long evolutionary preservation. On the other hand, further characterization of RNA polymerases and their promoters which are responsible for transcription of satellite DNAs could contribute to the elucidation of mechanisms of expression within condensed constitutive heterochromatin.
